# Public Dental Service personnel facing a major health care reform in Finland

**DOI:** 10.1038/s41405-019-0012-1

**Published:** 2019-04-11

**Authors:** Eeva Widström, Hannele Tiira, Anders Tillberg

**Affiliations:** 1 Department of Clinical Dentistry, Arctic University of Norway, Tromsø, Norway; 2 National Institute for Health and Welfare (THL), Helsinki, Finland; 3 Metropolia University of Applied Science, Helsinki, Finland; 4 Public Dental Health Service Competence Centre for Northern Norway, Tromsø, Norway

## Abstract

**Objectives:**

A health care reform will replace the health care and social services centred on public provision with a market-oriented system and enhanced competition between public and private sectors. The aim was to ascertain Public Dental Services (PDS) changes personnel anticipated and how dental services in the new “public” undertakings could be made more cost-efficient.

**Materials and methods:**

An electronic questionnaire was sent to the Chief Dentists of a random sample of 12 PDS units in southern and northern Finland for distribution to their personnel; 71.0% responded.

**Results:**

Most respondents (64.3%) believed that their PDS unit would not change. However, 45.4% foresaw a merger with another unit. More dentists (51.2%) were aware of market- and competition-oriented organisational forms to be introduced in the public sector than dental hygienists (35.0%) and dental assistants (27.3%; *p* < 0.01). Only 12.4% thought of moving to the private sector. To increase cost-efficiency in the new system, a majority suggested improvement in preventive care (79.8%) and increased use of dental hygienists (75.7%). A smaller proportion suggested longer opening hours (23.1%), higher patient fees (17.9%) or more paying patients (12.4%).

**Discussion:**

Public sector employees had little knowledge and understanding of the coming reform and were badly prepared for competition with the private sector.

## Introduction

Finland has a long tradition of universal, tax-financed health care and social services centred on public provision. An impending reform will change the structure of organising, provision and financing of these services including dental care. In 2015, the Government proposed establishment of 18 counties as early as July 2017 and transfer of the responsibility for organisation of health care and social services from the existing almost 200 municipalities to the new counties as of 1 January 2019.^1^ Later, both the establishment of the county councils and the change of responsible service organisers have been postponed to January 2021.^2^ The planned reform is the biggest in Finnish health care in 50 years and it follows the global wave and international policy movement towards marketing and competition as a way of challenging the public services, today often seen as “inefficient and unresponsive”^3^ and politically out-dated. People have moved from the countryside to towns and cities and a large proportion of the municipalities have become too small to administer health and social services. In addition, care and service needs have changed as the proportion of elderly has grown in the population.^1^

Parts of the planned reform are copied from neighbouring Sweden, which in 2010 introduced a so-called Choice Reform in Health Care.^4^ Also, in Finland free choice between public and private services is planned. According to the Government’s proposal, the former health administrative offices in the old system will become purchasers. Hospitals and primary health services may become semi-autonomous “trusts” that sell their services and compete with the private sector for contracts with the purchasers.^2^ This will also apply to dental care. By introducing a provider-purchaser split and demanding the formation of business enterprises from the public care providers the Government hopes to increase efficiency and effectiveness and to improve cost control.^2^ Much of the planning during the first years (2015–2016) concentrated on defining the numbers of county councils to be established, their borders, the future hospital network and its new hierarchical structure.

Historically, a municipal school dental service offering free, tax-financed dental service for school children, started the Public Dental Service (PDS) in Finland in 1956. Since 1972, the PDS has been part of the primary health care system run in municipal health centres. Initially, only children and adolescents were covered, but in the 1980s young adults were given access to subsidised services in the PDS and, after that, access was slowly expanded to include middle-aged adults. In 2002, all age groups were given access to the PDS. Alternatively, adults could use the more expensive private sector dental services partially reimbursed by the Social Insurance Institution.^5^

Dental professionals often feel challenged by health care reforms, e.g. public dentists in Sweden had difficulty coping with changes in their work environment caused by a reform in the mid-1990s that aimed at a more cost-efficient PDS.^6^ In Finland, the public dentists strongly opposed the reform in 2002 that gave older adults access to the public services, mainly due to perceived lack of resources.^7^ In Switzerland, recent plans for a mandatory dental care insurance to improve population access and early use of dental services has faced opposition from dentists’ associations.^8^

Half of the 4200 dentists (47%),^9^ and slightly more than half (60%) of the 1700 dental hygienists^10^ and 3000 dental assistants^11^ are currently working in the PDS as a salaried work force. In the reform, their work contracts will be cancelled and they will have to find new employments in the county-owned undertakings, the regional “County Council Limited Companies” to be established, or in private companies or they have to start their own businesses. This means that about 6000 dental professionals are at risk and the occupants need to prepare themselves for a changed work environment.

## Aim

The study aimed to find out whether the personnel working in the PDS were aware of coming changes in their work environment and what kind of changes they anticipated. A second aim was to study how they thought the new county-owned undertakings (the former public dental services) could be made more cost-efficient in the future. Comparisons were made between personnel groups: dentists, dental hygienists and dental assistants.

## Method

Ethical approval for a study about working conditions, job demands and impending changes in the PDS was granted by the Ethical Board of the National Institute for Health and Welfare (THL) (THL; protocol excerpts 8/2014). This article is based on two questions about possible changes in the dental care provision system in connection with the planned health care reform. They were formulated based on what could be anticipated in 2016 and had structured answer options: “Will the impending health care reform introduce changes in the PDS unit (health centre) where you work?” and “The present Government emphasises increased cost-efficiency in all work places. How can this be achieved in your PDS unit (health centre)?”. Three statements from the basic questionnaire were also used: 1. “I would like to work in my present work place to the end of my career”, 2. “It is likely that I move to the private sector”, and 3. “The number of dentists, dental hygienists and dental assistants is sufficient in relation to the numbers of patients and their treatment needs (after the older adults have received access to the PDS)”. For all questions space was provided for open comments or explanations.

Of the 90 health centres in the selected areas in northern and southern Finland, 12 PDS units were randomly selected. A link to an anonymous electronic questionnaire was sent to the Chief Dentists of be further distributed to their employees (dentists, dental hygienists and dental assistants; 438 persons according to the chief dentists). Altogether 311 PDS persons, 129 dentists, 61 dental hygienists and 121 dental assistants responded. This was 71.0% of the total number of personnel in the 12 clinics. There were 24 male dentists, all other respondents were women.

All statistical analyses were performed using the IBM® SPSS® statistics 25 for Mac personal computer. Chi-squared was used for categorical variables to analyse differences between groups. A *p*-value of <0.05 indicated a statistically significant difference. All respondents did not answer all questions, thus there is some variation in the *n*-values in the tables.

## Results

### Anticipated changes in the work places

Although two-thirds (64.3%) of the respondents believed that their own PDS unit would not change in the reform, almost half of them (45.4%) thought that it could be merged with another unit (health centre) (Table 1). Dentists (51.2%) were statistically significantly more often aware that market- and competition-oriented organisation forms could be introduced in the public sector than the dental hygienists (35.0%) and the dental assistants (27.3%; *p* < 0.01). Almost half of the dentists (42.9%) and dental hygienists (48.3%) but only a quarter of the dental assistants (26.1%; *p* < 0.01) believed that part of the present tasks of the PDS could be privatised. A smaller proportion of the respondents (13.6%) was afraid that their clinic could be closed down (Table 1).

**Table 1:** Positive answers (yes, most likely and yes, possible) to the question “Will the impending Health Care Reform introduce changes in the PDS unit (health centre) where you work?” by personnel group and geographical region in number and proportion (per cent)

	Dentists	Dental hygienists	Dental assistants		All	North	South	
	*n* (%)	*n* (%)	*n* (%)	*p-*value	*n* (%)	*n* (%)	*n* (%)	*p*-value
My PDS unit will most likely not change	86 (66.7)	35 (58.3)	77 (64.7)	n.s.	198 (64.3)	138 (69.0)	60 (55.6)	*
My PDS unit will most likely be merged with another PDS unit	61 (48.0)	29 (48.3)	49 (41.1)	n.s.	139 (45.4)	80 (40.2)	59 (55.2)	*
My PDS unit will become a municipal business enterprise or a regional limited company	65 (51.2)	21 (35.0)	32 (27.3)	**	118 (38.4)	62 (31.3)	56 (52.3)	**
Part of the activity in my PDS unit will be privatised	54 (42.9)	29 (48.3)	31 (26.1)	**	114 (37.5)	73 (36.7)	41 (38.7)	n.s.
My PDS unit will be closed down	18 (14.2)	9 (15.0)	14 (11.8)	n.s.	41 (13.6)	23 (11.5)	18 (16.8)	n.s.

Valid proportions by respondent group, dentists: *n* = 126–129; dental hygienists: *n* = 60; dental assistants: *n* = 118–119; northern Finland: *n* = 198–200; southern Finland: *n* = 106–108

*n.s.* Not significant

*p < 0.05; **p < 0.01

In the north, where there are fewer private dental clinics, the trust in continuity and stability was greater than in the south (69.0% versus 55.6%; *p* < 0.05) (Table 1). Some respondents commented the questions saying that dental personnel were not told about the reform plans locally. A few more respondents made remarks saying that because commercial companies were not able to run at a loss, redundancies by notice were to be expected by the personnel if the PDS was going to be changed to a business enterprise.

### Working conditions in the present work place

Slightly more than half of the respondents, 59.0% of the dentists, 49.2% of the dental hygienists and 57.9% of the dental assistants (*p* = ns) responded that they would like to work in their present work place to the end of their careers. Dentists (19.7%) and dental hygienists (15.0%) were significantly more likely to consider moving to the private sector than the dental assistants (3.4%; *p* < 0.01). More than half of the respondents claimed that their PDS unit had too few dentists (62.9%), dental hygienists (61.1%) and dental assistants (57.9%) in relation to the number of patients and their treatment needs. About two-thirds (60.9%) of the respondents felt that task sharing between personnel groups worked well in their clinic. Dental hygienists (50.0%; *p* < 0.05) were, however, statistically significantly less satisfied (Table 2).

**Table 2:** Responses to the following statements “I wish to work in my present work place/clinic to the end of my career”, “I will probably move to the private sector”, “Task sharing between personnel groups works well in my work place/clinic”, “The number of dentists/dental hygienists/dental assistants in relation to numbers of patients is on the right level”

	Dentists	Dental hygienists	Dental assistants		All	North	South	
	*n* (%)	*n* (%)	*n* (%)	*p*-value	*n* (%)	*n* (%)	*n* (%)	*p*-value
Engagement with the present work place/clinic								
Wishes to work in the present work place/clinic to the end of career								
Yes	75 (59.0)	30 (49.2)	70 (57.9)		175 (55.4)	113 (56.5)	62 (56.9)	
Cannot say	19 (15.0)	11 (18.0)	18 (14.9)		48 (16.0)	25 (12.5)	23 (21.0)	
No	33 (26.0)	20 (32.8)	33 (27.2)	n.s.	86 (28.6)	62 (31.0)	24 (22.1)	n.s.
Thinks of moving to private sector								
Yes, sure	3 (2.4*)*	2 (3.3)	0 (0)		5 (1.6)	3 (1.5)	2 (1.9)	
Yes, possible	22 (17.3)	7 (11.7)	4 (3.4)		33 (10.8)	19 (9.5)	14 (13.1)	
No	102 (80.3)	51 (85.0)	115 (96.6)	**	268 (87.6)	177 (90.0)	91 (85.0)	n.s.
Opinions on personnel								
Task sharing works well in my work place/clinic								
Yes	82 (64.1)	30 (50.0)	83 (68.6)		185 (60.9)	123 (61.5)	72 (66.1)	
Cannot say	12 (9.4)	3 (5.0)	12 (9.9)		27 (8.1)	18 (9.0)	9 (8.3)	
No	34 (26.5)	27 (45.0)	26 (21.5)	*	87 (31.0)	59 (29.5)	28 (25.6)	n.s.
Number of dentists in the clinic								
Too few	90 (70.3)	33 (55.0)	77 (63.6)		200 (62.9)	129 (64.5)	71 (65.1)	
Satisfactory	36 (28.1)	24 (40.0)	39 (32.3)		99 (33.5)	67 (35.5)	32 (29.4)	
Too many	2 (1.6)	3 (5.0)	5 (4.1)	n.s.	10 (3.6)	4 (2.0)	6 (5.5)	n.s.
Number of dental hygienists								
Too few	76 (59.8)	38 (62.3)	74 (61.2)		188 (61.1)	123 (61.2)	65 (60.2)	
Satisfactory	48 (37.8)	23 (37.7)	44 (36.3)		115 (37.3)	75 (37.3)	40 (37.0)	
Too many	3 (2.4)	—	3 (2.5)	n.s.	6 (1.6)	3 (1.5)	3 (2.8)	n.s.
Number of dental assistants								
Too few	75 (59.1)	31(51.7)	76 (62.8)		182 (57.9)	114 (57.0)	68 (63.0)	
Satisfactory	50 (39.3)	25 (41.7)	43 (35.5)		118 (38.8)	83 (41.5)	35 (32.4)	
Too many	2 (1.6)	4 (6.6)	2 (1.7)	n.s.	8 (3.3)	3 (1.5)	5 (4.6)	n.s.

Valid proportions by respondent group, dentists: *n* = 127–129; dental hygienists: *n* = 60–61; dental assistants: *n* = 120–121; northern Finland: *n* = 200–201; southern Finland: *n* = 108–110

*n.s.*  Not significant

*p < 0.05; **p < 0.01

### Means to improve cost-efficiency in the future PDS

As can be seen in Table 3, a great majority of the respondents thought that changes in the clinical treatment of the patients e.g. improvement in emergency care (84.5%), periodontal treatment (79.8%) and the introduction of comprehensive treatment for all adults (75.2%), would improve cost-efficiency. The respondents thought that more continuing education (63.5%) for the personnel and better task sharing between personnel groups (60.5%) would be needed. Dental hygienists (68.3%) had most confidence in the beneficial effects of increasing the division of labour between the various professional groups. About half of the respondents, 54.2% in the south and 42.9% in the north (*p* = ns), thought that greater patient flow would help to increase income in the PDS. Longer opening hours (23.1%), catering for more paying patients (12.4%) and higher patient fees (17.9%) received little support from the respondents (Table 3).

**Table 3:** Answers to the question “The present Government emphasises increased cost-efficiency in all work places. How can this be achieved in your PDS unit (health centre)?” by personnel groups and geographical region

	Dentists	Dental hygienists	Dental assistants		All	North	South	
	*n* (%)	*n* (%)	*n* (%)	*p*-value	*n* (%)	*n* (%)	*n* (%)	*p*-value
Measures related to the performance management								
Giving the personnel continuing education aiming at a well-functioning working unit	78 (60.9)	39 (65.0)	78 (65.2)	n.s.	195 (63.5)	136 (68.3)	59 (54.6)	*
Making more use of the specialised dentists or employing more specialists	75 (58.6)	27 (45.0)	56 (47.1)	n.s.	158 (52.0)	111 (55.8)	47 (43.5)	*
Reorganisation of the activities using various performance management tools such as Lean#	55 (43.0)	29 (48.3)	39 (32.5)	n.s.	123 (40.2)	68 (34.3)	55 (50.9)	**
Introducing recalls for adult patients	37 (28.9)	17 (28.8)	26 (21.0)	n.s.	80 (26.2)	50 (25.1)	30 (28.3)	n.s.
Measures related to the treatment of patients								
More immediate treatment of dental emergencies	107 (83.6)	48 (80.0)	104 (86.7)	n.s.	259 (84.5)	167 (83.5)	92 (85.2)	n.s.
Introducing comprehensive treatment for all age groups	92 (72.4)	47 (78.3)	89 (76.7)	n.s.	228 (75.2)	148 (75.9)	80 (74.1)	n.s.
Measures related to the quality of clinical work								
Increasing periodontal and preventive treatment for adult patients	108 (84.4)	50 (83.3)	87 (73.1)	*	245 (79.8)	162 (81.4)	83 (76.9)	n.s.
Improving the quality of clinical work	71 (56.8)	36 (60.0)	64 (54.2)	n.s.	171 (56.4)	114 (58.2)	57 (53.3)	n.s.
Measures related to improving the economy of the PDS unit								
Increasing patient flow	62 (48.4)	33 (55.0)	48 (41.0)	n.s.	143 (46.6)	85 (42.9)	58 (54.2)	n.s.
Higher patient fees	34 (26.4)	11 (18.6)	10 (8.4)	**	55 (17.9)	24 (12.0)	31 (29.0)	**
Catering for more paying patients	14 (10.9)	9 (15.3)	15 (12.6)	n.s.	38 (12.4)	26 (13.1)	12 (11.2)	n.s.
Staggering the opening hours of the clinics	25 (19.7)	17 (28.3)	29 (24.6)	n.s.	71 (23.1)	38 (19.3)	33 (30.6)	*
Measures related to the division of labour between personnel categories								
Increasing the work effort of dental hygienists in the clinical work	86 (67.7)	52 (86.7)	93 (78.8)	*	231 (75.7)	154 (77.8)	77 (72.0)	n.s.
Increasing the work effort of dental assistants in the clinical work and in motivating patients in home care	86 (67.2)	48 (80.0)	72 (61.0)	n.s.	206 (67.3)	127 (64.1)	79 (73.1)	n.s.
Increasing the division of work between dentists	71 (55.9)	41 (68.3)	72 (61.9)	n.s	184 (60.5)	110 (56.1)	74 (68.5)	*

Valid proportions by respondent group, dentists: *n* = 125–128; dental hygienists: *n* = 59–60; dental assistants: *n* = 118–120; northern Finland: *n* = 195–200; southern Finland: *n* = 106–108

*n.s.* Not significant

*p < 0.05; **p < 0.01

A third of the respondents (31.5%; 47 dentists, 32 dental hygienists and 19 dental assistants) provided comments on the statements in the open space reserved for this. Most of the comments (*n* = 61) complemented the statements about preventive treatment, quality of clinical treatment, task division between personnel groups and about “Lean management” aiming to improve organisations’ efficiency and quality through small incremental changes in processes; this was a popular subject for continuing education at the time. Typical comments were: “There should be more dental hygienists than dentists in the clinics” and “the patients should be obliged to perform home care for example with a written consent.” In addition, 19 respondents highlighted the perceived shortage of personnel in their PDS unit and eight respondents felt a need for “investments in the well-being of the personnel”. Ten respondents longed for better leadership in their PDS unit.

## Discussion

In oral health care, private provision of services has been greater than in the general health care. The PDS has catered for almost all the young (<18 years) and about half of the adults who visited a dentist in the course of a year (about 50%) and the private sector the other half.^12^ The private sector has provided regular and more expensive care and recall appointments whereas the public sector, with low (set) fees, has provided adults with more emergency services and long waiting lists for non-emergency care.^12^

When this survey was conducted in the autumn of 2016, there was little concrete information on details of the health care reform and the coming changes except that the principle of a purchaser provider split and increased competition with the private sector would be introduced. This might have contributed to the high response rate to this study. However, the responding dentists made up just above 12% of all public dentists in the areas considered. Corresponding percentages for dental hygienists and dental assistants can be estimated to be in the same order of magnitude and therefore the results must be interpreted with some caution. Because of the vague plans for the future dental care, it was not surprising that most dental professionals participating in this study (64%) believed—or hoped—that their own PDS unit would not need to change. It was also obvious that PDS employees did not wish to move to the private sector.

In the new system, county councils will choose or select the oral health care producers either using a tendering process or (more likely) by setting certain criteria to be fulfilled by the applicants. The suppliers will be paid by means of capitation for all or most dental care of children and adolescents and for some basic care of adults. For as yet undefined subsidised treatment measures, adults will pay the same fees regardless of the treatment sector.^2^ These are, however, most likely to be fewer and more expensive than before. For non-subsidised treatment measures, both the public and the private sectors will be free to set fees and this part of the work will be critical for the economy of both sectors. It seems obvious that to survive, the new county-owned undertakings (former PDS organisations) will need to take a closer look at the numbers of dental personnel to be employed as adults’ use of services may drop due to higher patient fees. Among other things, remuneration of the staff and opening hours of the clinics probably will need to be reviewed too, as private companies are much better prepared for competition.^13,14^

In principle, dental care can be made more cost-efficient by decreasing inputs (personnel and other costs) and increasing outputs (income from paying patients and rapid flow of capitation patients). However, in order to increase cost-efficiency, all personnel categories in this study suggested, in the first place, measures to be taken in patients’ clinical treatment such as more efficient emergency care and increased periodontal and preventive care and improved division of tasks between personnel groups, aspects they were most familiar with. It is true that regarding periodontal care and oral hygiene, the quality of Finnish dental care has been inadequate.^15,16^ However, it is more interesting that few respondents suggested longer opening hours, to facilitate working adult patients or increasing the numbers of paying patients and raising fees, probably because these strategies would be inconvenient for the personnel.^14,17^ Thus, the suggestions of stronger leadership in the new county-owned undertakings (former PDS organisations) by a number of respondents probably were wise as it was apparently difficult for public employees to envisage the business side of the enterprise.

Recently, the Finnish Nurses Association conducted a questionnaire study among their members about the planned health care reform. The results showed that even the nurses anticipated great changes in their work in the future. Few respondents believed that the reform would fulfil its goals: improve access to care (21%) and inhibit cost increases (17%). Most respondents (87%) thought that free choice between public and private treatment providers would create new problems and 58% believed that the reform would lead to fewer job openings.^18^

Big reforms influencing people’s terms and working conditions cause stress and are poorly received by the personnel involved.^5–7^ This study indicates that dental personnel in the PDS was not much involved in the planning of the new county-owned dental organisations where they are most likely to find work. It has to be mentioned that during recent years, private practitioners have increasingly been selling their practices to dental chains and are becoming employees of the chains, to be better prepared for competition from the public sector of the future contracts with the purchasers.

## Conclusions

Dental personnel expected major changes in their working conditions, especially in southern Finland where the private sector has a greater market share than in the northern parts of the country. Dentists and dental hygienists seemed to be more aware of possible market-oriented changes than dental assistants. Public sector employees seemed to have little understanding of how the work could be made more cost-efficient and were thus badly prepared for increased competition with the private sector.

## References

[1] Ministry of Health and Welfare. *Social services, Health Care and Regional Government Reform Put Before Parliament*. https://alueuudistus.fi/en/frontpage. Accessed 2 March 2017.

[2] Finnish Government. *Regional Government, Health and Social Services Reform*. https://www.regionalreform.fi. Accessed 15 October 2018.

[3] Light D (2003). Universal health care: lessons from the British experience. Am. J. Public Health.

[4] Dahlgren D (2014). Why public health services? Experiences from profit-driven health care reforms in Sweden. Int J. Health Serv..

[5] Widström E, Agustsdottir H, Byrkjeflot LI, Pälvärinne R, Boge Christensen L (2015). Systems for provision of oral health care in the Nordic countries. Tandlaegebladet.

[6] Bejerot E. Dentistry in Sweden: Healthy Work or Ruthless Efficiency? Thesis. Arbetslivsinstitutet. Arbete och hälsa 14 (CM gruppen, Solna, 1998).

[7] Widström E, Tiira H, Alestalo P, Pietilä I (2010). Terveyskeskushammaslääkärit uuden edessä. [English Summary: Public dentists’ experiences of a major oral health care reform]. Suom. Hammaslääk..

[8] di Bella Enrico, Leporatti Lucia, Montefiori Marcello, Krejci Ivo, Ardu Stefano (2017). Popular initiatives in 2014–2016 call for the introduction of mandatory dental care insurance in Switzerland: The contrasting positions at stake. Health Policy.

[9] Personal communication with the Finnish Dental Association. Numbers of public dentists in Finland in 2016. Accessed 27 May 2017.

[10] Personal communication with the Association of Dental Hygienist in Finland. Numbers of dental hygienists in Finland in 2016. Accessed 27 May 2017.

[11] Tilastokeskus. *Kuntasektorin palkkatilasto 2016**(Statistics Finland. Statistics on wages among municipal employees in 2016)*. www.tilastokeskus.fi. Accessed 27 May 2017.

[12] Widström E, Komu M, Mikkola H (2013). Longitudinal register study of attendance frequencies in public and private dental services in Finland. Community Dent. Health.

[13] Kottonen A (2017). Ei vain sanoja paperilla (Not. only words a Pap., Finn.). Suom. Hammaslääk..

[14] Widstrom E, Mikkola H (2013). Industry structures in private dental markets in Finland. Community Dent. Health.

[15] Koskinen S., Lundqvist A., Ristiluoma N. *Terveys, toimintakyky ja hyvinvointi Suomessa 2011* (Health, functional capacity and welfare in Finland in 2011, English summary). Terveyden ja hyvinvoinnin laitos (THL), Raportti 68. Helsinki: THL 2012. https://www.julkari.fi/bitstream/handle/10024/90832/Rap068_2012_netti.pdf.

[16] Widström E, Linden J, Tiira H, Seppälä TT, Ekqvist M (2015). Treatment provided in the Public Dental Service in Finland in 2009. Community Dent. Health.

[17] Widström E, Kemppinen M, Linden J (2016). Johtavat hammaslääkärit uskovat tehostamismahdollisuuksiin sotessa. (Chief dentists believe in increasing efficiency in the PDS in the impending major social and health care reform in Finland, English Summary). Suom. Hammaslääk..

[18] Sairaanhoitajaliitto. *Jäsenkysely sote uudistuksesta 2017 (Association of Finnish Nurses. Questionnaire study of the health care reform among members)*. https://sairaanhoitajat.fi/2017/kysely-sairaanhoitajat-epailevia-sote-uudistuksesta/. Accessed 15 November 2017.

